# “God speaks to me through a dove”. The evidence of clozapine in treatment-refractory psychosis

**DOI:** 10.1192/j.eurpsy.2024.1539

**Published:** 2024-08-27

**Authors:** B. Rodríguez Rodríguez, P. Marqués Cabezas, M. Queipo de Llano de La Viuda, N. Navarro Barriga, G. Guerra Valera, M. B. Arribas Simón, C. Alario Ruiz, M. J. Mateos Sexmero, M. Fernández Lozano, P. Marínez Gimeno, M. Calvo Valcárcel, M. A. Andreo Vidal, M. P. Pando Fernández, A. Monllor Lazarraga, M. Ríos Vaquero, G. Lorenzo Chapate, L. Rojas Vázquez, C. De Andrés Lobo, T. Jiménez Aparicio, M. D. C. Vallecillo Adame

**Affiliations:** ^1^Psiquiatría, Hospital Clínico Universitario de Valladolid, Valladolid; ^2^Psiquiatría, Hospital Clínico Universitario de Valladolid, Valladolidº, Spain

## Abstract

**Introduction:**

Clozapine is an atypical antipsychotic synthesised in 1958. It was withdrawn from the market in the 1970s due to the appearance of agranulocytosis, but was reintroduced due to strong evidence of its efficacy and superiority over other antipsychotics in treatment-resistant schizophrenia.

**Objectives:**

To describe the adequate response to clozapine in treatment-refractory psychosis.

**Methods:**

Review of the scientific literature based on a relevant clinical case.

**Results:**

A 16-year-old woman was admitted to a psychiatric inpatient unit for psychotic symptoms and behavioural disorders. She lives with her father and older sister; she has not been in contact with her mother, who lives in another country, for several years. She attends secondary school, with poor academic performance. Maternal diagnosis of schizophrenia. She started using cannabis two years ago, with a progressive increase up to 20 grams per week. He reports the onset of a feeling of strangeness a year ago, with progressive isolation in his room, referring to delirious ideation of harm towards classmates and people from his town, self-referentiality and delirious interpretations of religious mystical content (“God speaks to me through a dove”). He comments on the phenomenon of theft and thought-reading. Soliloquies and unmotivated laughter are observed.

**Conclusions:**

Treatment was started with risperidone, progressively increasing the dose up to optimisation, without achieving a decrease in positive symptoms, but with the appearance of excessive sedation and sialorrhoea. It was combined with aripiprazole up to 20mg, maintained for a couple of weeks, without significant clinical improvement. Given the failure of two lines of therapy, it was decided to change to clozapine up to a dose of 75mg, with adequate tolerance and response, achieving a distancing of the delirious ideation. Regular haematological controls were performed, with no alterations in haemogram or troponins.

**Disclosure of Interest:**

None Declared

